# Single-Cell Transcriptome Analysis Profile of Meniscal Tissue Macrophages in Human Osteoarthritis

**DOI:** 10.1155/2020/8127281

**Published:** 2020-07-28

**Authors:** Jingbin Zhou, Zhihong Zhao, Chen He, Feng Gao, Yu Guo, Feng Qu, Yujie Liu

**Affiliations:** ^1^Medical School of Chinese PLA, Beijing 100853, China; ^2^Chinese National Institute of Sports Medicine, Beijing 100061, China; ^3^Department of Orthopedics, Beijing Second Hospital, Beijing 100031, China; ^4^Department of Foot and Ankle Surgery, Beijing Tongren Hospital Affiliated to Capital Medical University, Beijing 100730, China

## Abstract

Osteoarthritis (OA) has long been considered as a degenerative disease, but growing evidence suggests that inflammation plays a vital role in its pathogenesis. Unlike rheumatoid arthritis and other autoimmune diseases, inflammation in OA is chronic and, in relatively low grade, mainly mediated by the innate immune system, especially macrophages. However, due to its low abundance, there is a lack of systematic studies on macrophages in the OA condition. Here, we have used single-cell RNA sequencing analysis to gain insight into the heterogeneity and functional specialization of human knee macrophages. We also compared the gene expression profiles of macrophages in healthy people and OA patients and found the characteristic changes of special macrophages in the OA knee. We believe that this in-depth understanding of the basis of OA inflammation will bring hope for the development of new therapies.

## 1. Introduction

Osteoarthritis (OA) is a common joint degenerative disease, characterized by the progressive destruction of articular cartilage, involving the subchondral bone and the synovium. However, the pathological process of OA, especially the molecular mechanism, still needs to be understood [1]. Traditionally, OA has been considered as a noninflammatory disease [2]. With the deepening of research, more and more studies have shown that the immune system plays an important role in the progress of OA and is closely related with the pathological changes of articular cartilage and synovium [3, 4], which began to define OA as a low-grade inflammation state and explores the pathogenesis of OA from a perspective of the immune system [5, 6]. Therefore, a comprehensive study of its related immune cells and subtypes is required.

The knee is the most common site of OA and believed to be the price that humans pay for walking upright. The knee OA is initiated with the wear and tear of articular cartilage and the inevitable inflammation [7, 8], so the focus of research should be on the inflammation regression and the process of tissue repair. In these processes, tissue macrophages in the knee are crucial [9]. With strong plasticity, macrophages can be divided into M1 subgroups that trigger inflammation and release a large number of proinflammatory factors or M2 subgroups that suppress inflammation, reshape tissues, and release anti-inflammatory factors and growth factors [4, 10]. However, recent studies show that M1 and M2 subpopulations may only be two extreme types of differentiation and macrophages may be reprogrammed according to their microenvironment [11]. The latest animal experiments have found a special type of macrophage that exists in healthy knee joints, which not only forms a structural barrier but can also digest and remove neutrophils in rheumatoid arthritis to form joint immune barrier [12]. However, the dynamic changes of macrophages in the human OA are unclear, and their gene expression characteristics need further investigation.

In this study, we use the newly released single-cell sequencing data of knee tissues from healthy people and OA patients to identify macrophage subsets and characterize the differential gene expression involved in the OA pathogenesis. Our data show the diversified characteristics of specific cell types and explore the potential transition process of macrophages in the healthy and OA knee.

## 2. Methods

### 2.1. Single-Cell mRNA Sequencing Data

Data from Sun et al. [13] are downloaded from the Gene Expression Omnibus (GEO) dataset and are accessed at GSE133449. These scRNA data were from the cells isolated from the meniscus with the synovium specifically removed. Of the total 6833 cells, 3577 cells were obtained from people without OA, and the other 3256 cells were obtained from patients with OA.

### 2.2. Single-Cell mRNA Sequencing Analysis Tool

The volcano plots for the differential gene expression (DEG) study were drawn using the R package ggplot2. Other plots were drawn using the tools in the R package Seurat.

### 2.3. Single-Cell mRNA Sequencing Analysis

Using Seurat, we determined 15 principal components (PC) and performed dimensionality reduction and cluster analysis with a resolution parameter of 0.5. A differential expression analysis was performed on each cluster, and the results were visualized using Uniform Manifold Approximation and Projection (UMAP). Nonchondrocyte clusters were subclustered, and Feature plot was used to define the gene expression patterns in the clusters. A heat map was employed to characterize the top 10 genes in the clusters. Dot plots were used to demonstrate the expression pattern and level of expression of specific genes. The enhanced volcano plots displayed the DEG between two clusters of cells, in which we enter the normalized gene count converted by Log_2_ to obtain DEG. For DEG testing, the value is <0.01 and is considered DEG.

## 3. Results

After strict quality control and data collation, 3577 cells from healthy people and 3256 cells from OA patients were retained for subsequent analysis. In order to solve the heterogeneity between the healthy and OA cells and remove the batch effect in between, we used the SCTransform method [14] to integrate and correct the data from the healthy and OA groups before the principal component analysis (PCA).

**Figure 1:**
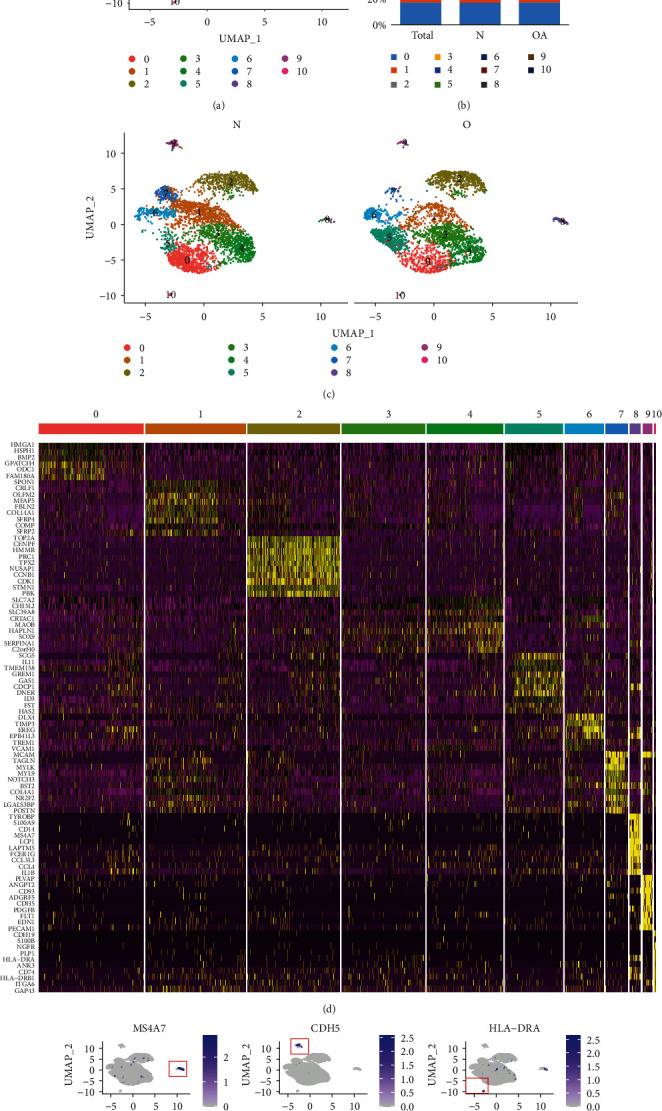
Single-cell RNA-seq analysis of cell types in the knee of healthy and OA patients. (a) UMAP plot of 6833 single cells from the human knee depicting 11 major cell types. Colours correspond to the clusters 0 to 10. (b) Bar plot shows frequency of each cell type in total, healthy, and OA groups. (c) Split UMAP plot depicting the clusters from healthy (N) and OA patients (O). (d) Heat map indicates the top 10 marker genes for each cell type. (e) Expression distribution of the top marker genes for the nonchondrocytes projected onto the UMAP plot: macrophages (MS4A7), endothelial cells (CDH5), and monocytes (HLA-DRA).

To study the heterogeneity of knee cells, we used the selected PC load as input and clustered cells with UMAP. After PCA clustering with a resolution of 0.5, a total of eleven hypothetical cell clusters were generated, including 8 chondrocyte-based populations and 3 non-chondrocyte-based populations (Figure 1(a)). The clusters in the healthy and OA groups are comparable, although the proportions differ (Figures 1(b) and 1(c)). Each cell type has been successfully annotated with known marker genes (Figures 1(d) and 1(e)). As the chondrocyte populations have been analysed in previous articles, we are interested here in three nonchondrocyte populations, namely, MS4A7+ macrophages (8), VE-cadherin+ (CDH5) endothelial cells (9), and HLA-DRA+ monocytes (10).

**Figure 2:**
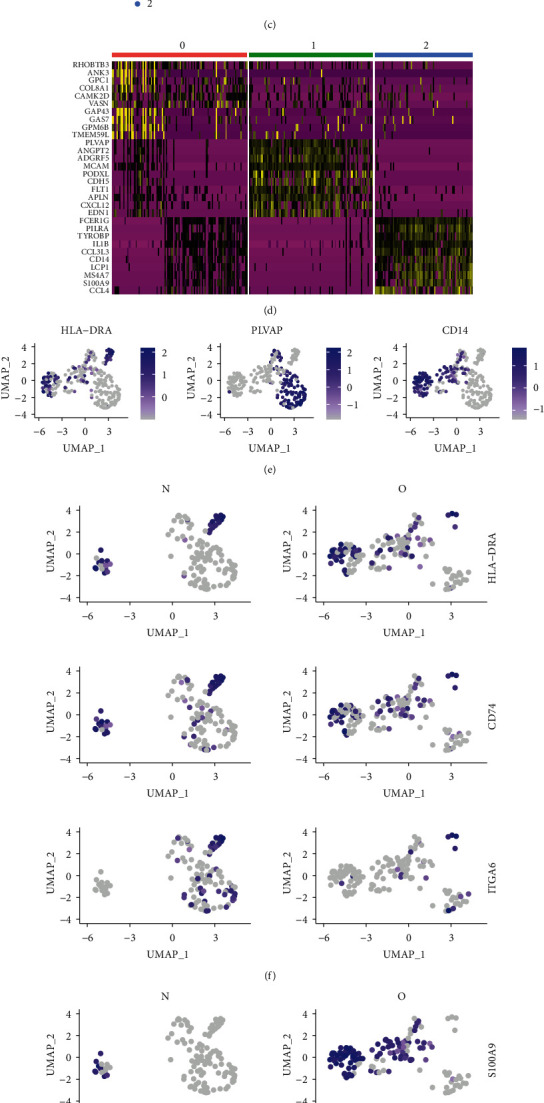
Single-cell RNA-seq analysis of nonchondrocytes from healthy and OA patients. (a) UMAP plot of 259 single cells from the human knee depicting 3 nonchondrocyte cell types. (b) Bar plot shows frequency of each cell type in the healthy and OA groups. (c) Split UMAP plot depicting the nonchondrocyte clusters from healthy (N) and OA patients (O). (d) Heat map indicates the top 10 marker genes for each cell type. (e) Expression distribution of the top marker genes for the nonchondrocytes projected onto the UMAP plot. (f) Expression distribution of top markers in the monocyte cluster. (g) Expression distribution of top markers in the macrophage cluster.

To further examine the molecular characteristics of nonchondrocytes at the single-cell level, we conducted population analysis of these cells in the healthy group and the OA group (Figure 2(a)). 120 nonchondrocytes from healthy people and 139 nonchondrocytes from OA patients were analysed. In nonchondrocytes, the number of macrophages in the OA group was much higher than that in the healthy group (Figures 2(b) and 2(c)). Macrophages (2), endothelial cells (1), and monocytes (0) are also reannotated with known marker genes (Figures 2(d) and 2(e)). The OA group showed less HLA-DRA-, CD74-, and ITGA6-expressing monocytes compared to the healthy group (Figure 2(f)), while there were much higher proportions of S100A9-, CD14-, and MS4A7-positive cells in the healthy group (Figure 2(g)).

**Figure 3:**
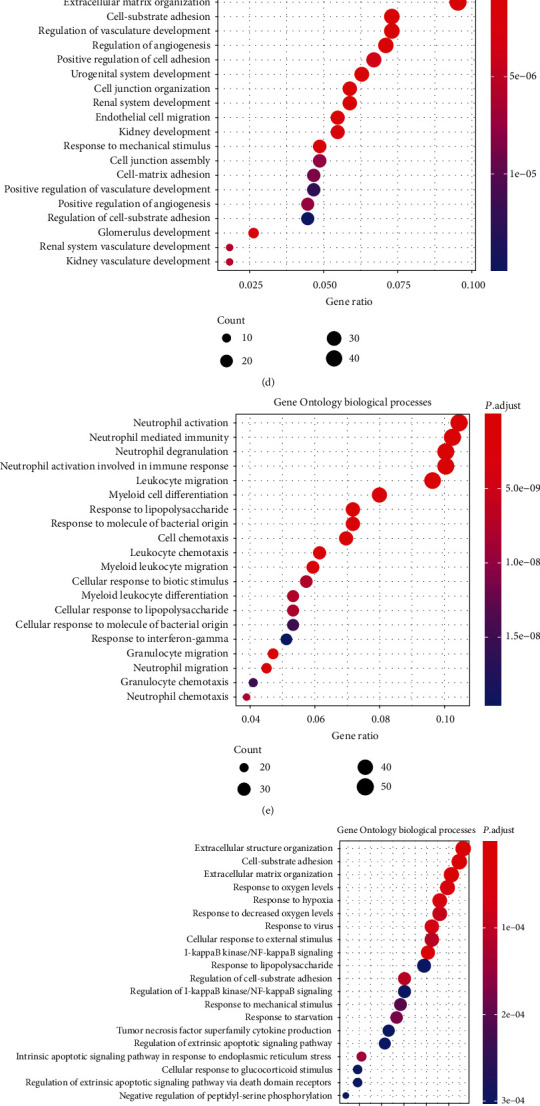
Comparison of DEG of the nonchondrocyte cluster from healthy and OA patients. (a) Volcano plot comparing the gene expression between healthy and OA monocyte clusters. (b) Volcano plot comparing the gene expression between healthy and OA endothelial cell clusters. (c) Volcano plot comparing the gene expression between healthy and OA macrophage clusters. Each plot represents one gene. Threshold of Log_2_ fold change has been set as 0.3. (d) Dot plot showing the enrichment of Gene Ontology biological processes in the upregulated monocyte DEG between healthy and OA tissues. (e) Dot plot showing the enrichment of Gene Ontology biological processes in the downregulated monocyte DEG between healthy and OA tissues. (f) Dot plot showing the enrichment of Gene Ontology biological processes in the upregulated endothelial cell DEG between healthy and OA tissues. (g) Dot plot showing the enrichment of Gene Ontology biological processes in the upregulated macrophage DEG between healthy and OA tissues.

To study the changes of nonchondrocytes in the OA state systematically, we compared the gene expression differences between the three cell types in healthy and OA states, including monocytes (Figure 3(a)), endothelial cells (Figure 3(b)), and macrophages (Figure 3(c)). It is clear that monocytes in the OA group tend to express activation markers such as IL1beta, CXCL5, and TYROBP, while the macrophages in the OA downregulate both M1 marker such as SIGLEC1 and M2 marker such as CSF2, indicating that macrophages in the OA state polarize towards directions other than the M1/M2 division. To further analyse the up- or downregulated gene expression in the monocytes, endothelia, and macrophages between healthy and OA tissues, we classified the DEGs using GO enrichment and focused on the biological processes of those genes (Figures 3(d)–3(g)).

**Figure 4:**
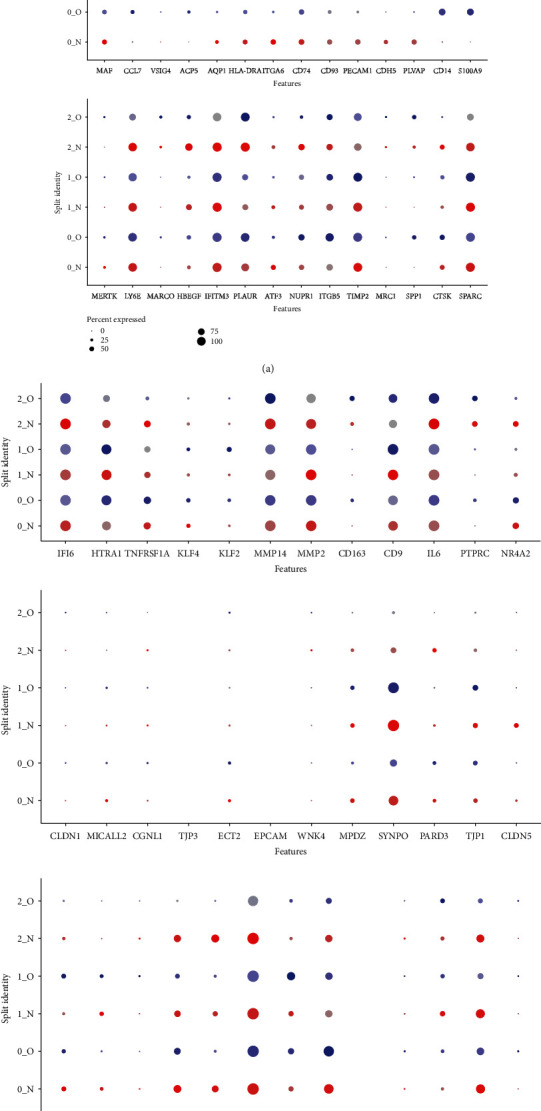
Special features of barrier macrophages in the OA tissue. (a) Dot plots demonstrating the expression pattern and level of expression of M1 or M2 genes. Colour intensities show the expression level of the indicated gene. (b) Dot plots demonstrating the expression pattern and level of expression of barrier macrophages. (c) CX3CR1 expression.

In order to clearly analyse the differences in gene expression profile, we use dot plots to show the proportions and intensities of different cells in different states (Figure 4(a)). Unexpectedly, macrophages in the OA state are more inclined to express the M2 markers, which is contrary to the concept that M2 macrophages promote the process of tissue repair [15].

Recent studies have shown a special kind of barrier macrophages in the outer layer of healthy knee synovium [11, 12]. These macrophages express certain characteristic molecules of epithelial cells, which not only form a tightly connected structural barrier but also digest and remove neutrophils in rheumatoid arthritis, forming an immune barrier in the synovium of the joint. However, the dynamic changes of these macrophages in the human OA remain unknown. Therefore, we compared the gene expression differences of barrier macrophage characteristic genes between the healthy and OA macrophages (Figure 4(b)). The tight junction genes of barrier macrophages are not unexpectedly expressed on endothelial cells. However, the expression of CX3CR1 is missing in the meniscal tissue macrophages (Figure 4(c)). As the murine barrier-forming synovial macrophages have been identified as CX3CR1+, this suggests a different feature in the meniscus. Nevertheless, the expression of these genes on macrophages of healthy tissues is much higher when compared to that of the OA tissues, indicating a correlation of these macrophages to the human OA pathogenesis.

**Figure 5:**
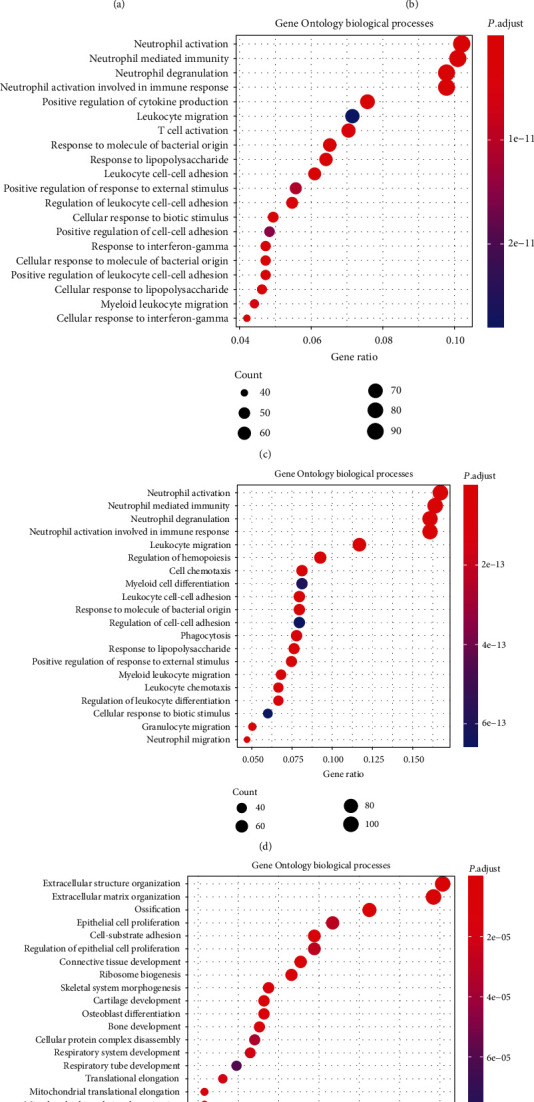
Gene expression comparison between monocytes and macrophages in the OA tissue. (a) Volcano plot comparing the gene expression between monocytes and macrophages in the healthy tissue. (b) Volcano plot comparing the gene expression between monocytes and macrophages in the OA tissue. (c) Dot plot showing the enrichment of Gene Ontology biological processes in the downregulated DEG between monocyte-like cell and macrophage in the healthy tissue. (d) Dot plot showing the enrichment of Gene Ontology biological processes in the downregulated DEG between monocyte-like cell and macrophage in the OA tissue. (e) Dot plot showing the enrichment of Gene Ontology biological processes in the upregulated DEG between monocyte-like cell and macrophage in the OA tissue.

In addition, we also compared the gene expression differences between monocytes and macrophages. In healthy tissues, macrophages demonstrate the typical genes of macrophages as well as the feature genes of monocytes, suggesting that monocytes might be present as the potential precursors of macrophages. In the OA cells, monocytes upregulate even more activation genes, such as CD44, CXCL1, and TYROBP, indicating a transformation of these monocytes in OA (Figures 5(a) and 5(b)). The DEGs have been classified with GO enrichment (Figures 5(c)–5(e)).

## 4. Discussion

Our research reveals the single-cell gene expression characteristics and transition process of meniscal resident macrophages in the knee and describes the transcriptional dynamics of macrophages during the OA process. Our data found that resident macrophages exhibit special characteristics that differ from the M1/M2 subtypes and adapt to their immune niche.

Macrophages in joints are normally under the steady state. When joint injury or aging stress leads to cell apoptosis, DAMP produced by the breakdown of chondrocytes and extracellular matrix can activate and differentiate macrophages into the M1 subpopulation through the canonical pathway [16], which upregulate the release of inflammatory factors and aggravate OA. In contrast, the use of rapamycin to inhibit mTORC1 or specific deficiency of mTORC1 in macrophages can polarize the cells to M2 macrophages, which engulf dead cells, accelerate the repair process, and inhibit synovial inflammation [9]. One might expect that M2 or wound healing macrophages might be more predominant in OA-damaged meniscus. However, these gene expression studies were based on a large number of RNA samples and only provide a virtual average of the cell mixture. Our current single-cell study can provide molecular differentiation of all cell types within a complex composition, which helps to improve the understanding of homogeneity and discover the complexity and transitional nature of macrophages in the OA joints.

The latest animal studies have shown that healthy knee macrophages express certain characteristic molecules of epithelial cells, forming a structural barrier with tight junctions. In a mouse model of rheumatoid arthritis, the barrier layer undergoes functional remodelling, which loses the barrier function of macrophages [12]. Using single-cell sequencing technology, they analysed specific genes of barrier macrophages in mice. The macrophages/monocytes in this study were from the meniscus and not the synovium. Current data indicate that although some specific genes expressed by barrier macrophages can be found, meniscal tissue macrophages display different characteristics from murine barrier-forming synovial macrophages. These features have undergone tremendous changes under OA conditions.

Different from the bulk-seq method, single-cell transcriptional research can make a molecular distinction between all cell types, which helps to improve the understanding of histological identity and decipher why adjacent cells make different differentiation decisions during development [17]. Here, we focus on the presence of macrophages in the joints and provide their connection with OA pathogenesis, which may provide potential targets and pathways for OA treatment.

## Data Availability

Data from Sun et al. (13) are downloaded from the Gene Expression Omnibus (GEO) dataset and are accessed at GSE133449. All other data supporting the findings are available from the corresponding author on reasonable request.
